# Tuft-cell-derived IL-25 regulates intestinal ILC2 in response to *Brucella* infection

**DOI:** 10.3389/fimmu.2026.1732274

**Published:** 2026-02-06

**Authors:** Kaiyu Shang, Na Chen, Tingting Tian, Huidong Shi, Juan Shi, Xinxin Qi, Yuejie Zhu, Fengbo Zhang

**Affiliations:** 1 Department of Clinical Laboratory, The First Affiliated Hospital of Xinjiang Medical University, Urumqi, Xinjiang, China; 2 Reproductive Medicine Center, The First Affiliated Hospital of Xinjiang Medical University, Urumqi, Xinjiang, China

**Keywords:** *Brucella* infection, IL-25, ILC2, intestinal immunity, tuft cells, tuft cell-ILC2 circuit

## Abstract

**Introduction:**

*Brucella* is a zoonotic pathogen capable of invading the host through the intestinal mucosa. However, the immune mechanisms underlying intestinal infection remain poorly understood. Tuft cells are specialized chemosensory epithelial cells in the intestine that can detect pathogen invasion and secrete IL-25, subsequently activating type 2 innate lymphoid cells (ILC2s) and playing a critical role in anti-parasitic immune responses. Nevertheless, whether the tuft cell-ILC2 circuit participates in immune responses against bacterial infections remains unclear. This study aimed to investigate the dynamic changes of tuft cells and ILC2s following *Brucella* infection, with a particular focus on elucidating the regulatory role of IL-25 in this process.

**Methods:**

Thirty-six mice were divided into six groups: normal control (NC), isotype control (IC), infection groups at different time points (3, 7, and 14 days post-infection, designated as Inf-3d, Inf-7d, and Inf-14d, respectively), and an IL-25 blockade group (IL-25 Blk-7d, in which mice received anti-IL-25 neutralizing antibody treatment prior to *Brucella* infection and were analyzed at 7 days post-infection). In this study, immunofluorescence assay, flow cytometry, and Western blot were employed to detect the dynamic changes of tuft cells and ILC2s in intestinal tissues, as well as to determine the expression levels of pathway-related proteins.

**Results:**

The results showed that the numbers of tuft cells and ILC2s in the mouse intestine increased following *Brucella* infection, peaking at 7 days post-infection. Pretreatment with IL-25 neutralizing antibody significantly suppressed the proliferation of these two cell populations. Western blot analysis further confirmed that the expression levels of tuft cell-associated proteins (PO2F3, DCLK1, and IL-25) and ILC2-associated proteins (GATA3 and IL-13) were upregulated in infected intestinal tissues, whereas IL-25 blockade treatment inhibited the expression of these proteins. Correlation analysis revealed a remarkably strong positive correlation between the proportions of tuft cells and ILC2s, and this correlation was completely abolished following IL-25 neutralization.

**Discussion:**

These findings confirm that the activation of the tuft cell-ILC2 circuit is dependent on the regulatory role of IL-25. These findings extend the antimicrobial role of the tuft cell-ILC2 circuit beyond parasitic immunity and identify IL-25 as an essential regulatory mediator in antibacterial defense at the intestinal mucosa.

## Graphical Abstract

**Figure:**
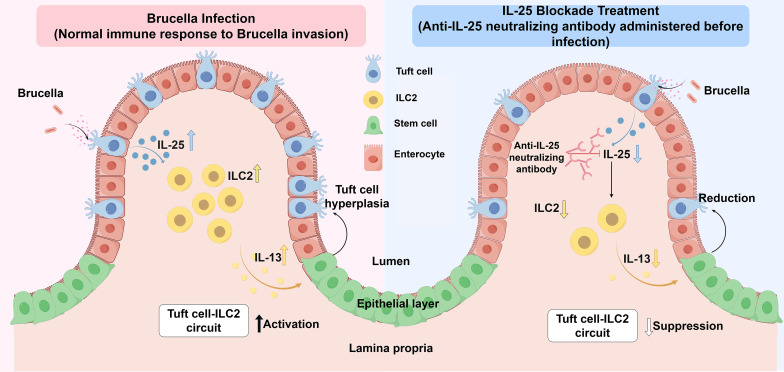
Created using Figdraw 2.0

## 1 Introduction

Brucellosis is a zoonotic systemic chronic infectious disease caused by bacteria of the genus *Brucella*, which poses enormous threats to livestock production and human health (1, 2). According to the latest research estimates, there are 2.1 million new cases of brucellosis globally each year, four times higher than previously reported (3).*Brucella* can invade the host through various routes, with the gastrointestinal tract being one of the important portals of entry. Humans are primarily infected with *Brucella* through consumption of inadequately pasteurized dairy products or undercooked meat (4, 5). Although the intestinal invasion route of *Brucella* has been established, the molecular mechanisms by which the host intestinal mucosal immune system recognizes and responds to *Brucella* infection remain unclear. Elucidating these mechanisms will provide us with a deeper understanding of the pathogenic mechanisms of this pathogen and help develop effective prevention and treatment strategies against brucellosis.

The intestinal epithelium not only serves as a physical barrier but also functions as a sentinel capable of detecting and responding to microbial threats. Tuft cells are a specialized type of chemosensory cells recently discovered in the intestinal epithelium, characteristically expressing the marker molecule Dclk1 (6–8). Studies have found that tuft cells can sense changes in the intestinal microenvironment and recognize pathogens through taste receptors and other chemoreceptors (9, 10). Once activated, tuft cells can secrete multiple cytokines, among which interleukin-25 (IL-25) is one of their most important effector molecules (11, 12). As a member of the IL-17 cytokine family, IL-25 plays a central role in initiating type 2 immune responses. IL-25 can specifically activate type 2 innate lymphoid cells (ILC2s). Activated ILC2s secrete large amounts of type 2 cytokines such as IL-5 and IL-13, with IL-13 promoting goblet cell hyperplasia and mucus secretion, thereby facilitating parasite clearance. Numerous studies have confirmed that the tuft cell-ILC2 immune circuit plays an irreplaceable role in combating intestinal parasitic infections, and activation of this circuit is critical for parasite elimination (11–14).

Despite these well-established functions in anti-parasitic immunity, whether the tuft cell-ILC2 circuit also participates in host defense against bacterial infections, particularly intracellular pathogens, remains largely unexplored. Given that the intestinal mucosa serves as a primary portal of entry for *Brucella* and that tuft cells function as immune sentinels at mucosal surfaces, we hypothesized that oral *Brucella* infection activates the tuft cell-ILC2 circuit, with IL-25 serving as a key mediator of crosstalk between these two cell types. To test this hypothesis, we established a mouse model of oral *Brucella* infection and systematically characterized the temporal dynamics of tuft cells and ILC2s in the intestinal mucosa during early infection. Using IL-25 neutralizing antibody blockade, we further evaluated the contribution of this cytokine to the intestinal mucosal immune response against *Brucella*. This study extends the understanding of the tuft cell-ILC2 circuit from anti-parasitic immunity to bacterial infections and may identify IL-25 as a potential therapeutic target for brucellosis prevention and treatment.

## 2 Materials and methods

### 2.1 Experimental animals

Female BALB/c mice (6–8 weeks old, Specific Pathogen Free (SPF) grade, body weight 18–22 g) were purchased from the Laboratory Animal Center of Xinjiang Medical University. Inclusion criteria: healthy female mice aged 6–8 weeks with body weight between 18–22 g, normal activity, and no visible abnormalities. Exclusion criteria: mice with body weight outside the specified range, visible injuries, abnormal behavior, signs of illness, or poor coat condition were excluded from the study. Animals were housed under controlled conditions with temperature maintained at 22 °C ± 1 °C and relative humidity at 50% ± 5%. A 12-hour light/dark cycle was employed, with 3–4 animals per cage (15). Prior to experimentation, mice underwent a one-week acclimation period in the SPF environment. The study protocol received approval from the Animal Ethics Committee, and all procedures complied with China’s national regulations for animal protection. All experiments involving live *Brucella* were conducted at the Biosafety Level 3 (BSL-3) laboratory of Xinjiang Uygur Autonomous Region Center for Disease Control and Prevention (Xinjiang CDC), which holds China National Accreditation Service (CNAS) accreditation (Registration No. CNAS BL0099) for BSL-3 operations in accordance with CNAS-CL05:2009 and GB 19489:2008 national biosafety standards. The facility is equipped with negative pressure ventilation, HEPA-filtered air handling systems, and dedicated Animal Biosafety Level 3 (ABSL-3) housing. All operators completed specialized BSL-3 training. Personal protective equipment, including full-face respirators with protective coveralls, double gloves, and face shields, was worn during all procedures. *Brucella* culture and bacterial suspension preparation were performed exclusively in Type A2 biological safety cabinets. Oral gavage inoculation was conducted inside biological safety cabinets to prevent aerosol generation. Infected animals were housed in the ABSL-3 facility in individually ventilated cages with HEPA filtration throughout the infection period, with all cage changes and animal handling performed within biological safety cabinets. All contaminated materials, including animal carcasses and bedding, were double-bagged and autoclaved at 121 °C for 30 minutes prior to disposal according to medical waste regulations. Laboratory personnel underwent regular health surveillance and serological monitoring for *Brucella* exposure.

### 2.2 Bacterial strain

The experiment utilized *Brucella melitensis* strain 16M (ATCC 23456). Initial inoculation was performed on blood agar plates, followed by a 3-day incubation at 37 °C in an atmosphere containing 5% CO_2_. Following cultivation, bacteria were harvested and resuspended in sterile physiological saline (16).

### 2.3 Animal grouping and treatment

Thirty-six mice were divided into six groups (n=6/group): normal control (NC), isotype control (IC), infection groups at 3, 7 and 14 days post-infection (Inf-3d, Inf-7d, and Inf-14d, respectively), along with an IL-25 blockade group that received anti-IL-25 neutralizing antibody prior to *Brucella* infection and was analyzed at 7 days post-infection (IL-25 Blk-7d). For infection, mice received 0.1 mL bacterial suspension containing approximately 1×10^10 CFU of *Brucella* via oral gavage (17), while the control groups received an equivalent volume of sterile PBS. To neutralize IL-25 signaling *in vivo*, mice were intraperitoneally (i.p.) injected with Anti-Mouse IL-25 Antibody (Clone 2C3, MedChemExpress, China, Cat: HY-P990216) at a dose of 25 mg•kg^-1^ body weight (approximately 500 μg/mouse based on an average body weight of 20 g) 24 hours prior to infection. Mouse IgG1 kappa (MedChemExpress, China, Cat: HY-P99977) was administered at the same dose as an isotype control (18).

#### 2.3.1 Antibody validation and safety assessment

The anti-IL-25 neutralizing antibody dosage and administration route (i.p.) were selected based on previously published protocols demonstrating effective IL-25 neutralization in murine models (18–20). Following antibody administration, all mice were monitored daily for clinical signs of adverse reactions, including changes in general health status (activity level, grooming behavior, posture), body weight, injection site reactions (swelling, redness, or abscess formation), and signs of systemic toxicity (piloerection, hunched posture, labored breathing). No significant weight loss (**Supplementary Figure S4**), behavioral abnormalities, or signs of toxicity were observed in animals either treated with the anti-IL-25 neutralizing antibody (IL-25 Blk-7d group) or untreated as infected controls (Inf-7d group) throughout the experimental period, confirming the safety of the antibody administration protocol.

The efficacy of IL-25 neutralization was validated through quantification of tuft cell numbers (DCLK1+ cells), analysis of ILC2 populations, and evaluation of downstream signaling molecules (GATA3) expression. The significant reduction in these IL-25-dependent readouts in the IL-25 Blk-7d group compared to the Inf-7d group confirmed successful blockade of the IL-25 signaling pathway.

### 2.4 Sample collection

Mice from the NC, IC, Inf-3d, Inf-7d, Inf-14d and IL-25 Blk-7d were euthanized by CO_2_, with six mice per group. The euthanasia chamber holds 11.172 liters (28×21×19 cm), and the CO_2_ flow rate is set at 5.59 liters per minute, providing a 50% displacement (21). Small intestinal tissues were collected aseptically. Portions of these tissues were fixed in 4% paraformaldehyde for immunofluorescence detection (22), while the remaining tissues were utilized for flow cytometry analysis.

### 2.5 Multiplex immunofluorescence

#### 2.5.1 Paraffin section preparation and antigen retrieval

Paraffin sections were heated at 65 °C for 2 hours, deparaffinized in xylene, and rehydrated through a descending ethanol gradient (100%-I, 100%-II, 95%, 85%, 75%) to distilled water. Antigen retrieval was performed in sodium citrate buffer (pH 6.0, Solarbio, China) using microwave heating at medium power for 15 minutes, followed by natural cooling to room temperature.

#### 2.5.2 Immunostaining procedure

Multiplex immunofluorescence was performed using the Absin staining kit (Cat: abs50028). Endogenous peroxidase was blocked with 3% hydrogen peroxide for 10 minutes at room temperature in the dark, followed by serum blocking with goat serum (Zhongshanjinqiao, Cat: ZLI-9022) for 60 minutes and 30 minutes for subsequent rounds. For the first round, sections were incubated with rabbit anti-DCLK1 antibody (Proteintech, Cat: 21699-1-AP, 1:500) overnight at 4 °C, followed by HRP-conjugated secondary antibody (10 min, 37 °C) and TSA-520 fluorescent dye (10 min, 37 °C). After antigen retrieval and blocking, the second round employed rabbit anti-*Brucella* antibody (Bioss, Cat: bs-2229R, 1:200, 1 hour at 37 °C), followed by HRP-secondary antibody and TSA-650 fluorescent dye. Sections were counterstained with DAPI and mounted with anti-fade medium. Between each step, slides were washed three times with 1×TBST for 5 minutes each. Detailed antibody information is shown in **Supplementary Table S1**.

#### 2.5.3 Image acquisition and quantification

Images were acquired using a Nikon confocal microscope with identical settings across all samples. Tuft cells were quantified by counting DCLK1+ cells in at least 5 random high-power fields (HPF, 600×) per mouse from the jejunum region. Cell counting was performed manually in a blinded manner using ImageJ software with the Cell Counter plugin. Only cells with clear nuclear DAPI staining and strong cytoplasmic DCLK1 signal were counted. The average count of DCLK1+ cells per field was calculated for each mouse and used for statistical analysis.

### 2.6 Flow cytometry

#### 2.6.1 Intestinal cell isolation

Intestinal epithelial cells were isolated for tuft cell detection, and lamina propria cells were isolated for ILC2 analysis. Small intestines were harvested, cleared of adipose tissue, cut into 4–5 cm segments, and flushed with PBS. After vigorous washing in a 50 mL tube for 2 minutes, epithelial cells were dissociated by sequential incubation in pre-warmed (37 °C) dissociation solution 1 (1× Hanks buffer, 10 mM DTT, 30 mM HEPES) and dissociation solution 2 (1× Hanks buffer, 10 mM EDTA, 30 mM HEPES) at 250 rpm for 15 minutes each (23). The cell suspension was filtered through a mesh filter and centrifuged at 1050 g for 5 minutes to obtain epithelial cells.

For lamina propria cell isolation, EDTA-treated intestinal segments were washed with PBS (5 minutes), cut into <0.5 cm pieces, and digested in pre-warmed digestion solution (10% FBS, 5 mg•mL^-1^ DNase I, 5 mg•mL^-1^ Collagenase IV) at 250 rpm and 37 °C for 35 minutes (24). The digested tissue was filtered through a 75 μm nylon mesh, and cells were purified using a 40%/80% Percoll gradient centrifugation (1000 g, 20 minutes, minimal acceleration/deceleration). The interface layer containing lamina propria lymphocytes was collected, washed with PBS, and centrifuged at 800 g for 10 minutes.

#### 2.6.2 Flow cytometric analysis

Murine tuft cells were identified as viability dye negative, CD45^-^, EpCAM^+^, DCLK1^+^ cells. Since DCLK1 is an intracellular marker, cells were subjected to membrane permeabilization and fixation using the BD Cytofix/Cytoperm™ Fixation/Permeabilization Kit (BD, Cat: 554714) (25). Due to the lack of commercially available fluorophore-conjugated DCLK1 antibodies, indirect immunofluorescence was employed using an unconjugated primary antibody followed by CoraLite 488-labeled Goat Anti-Rabbit IgG (Proteintech, China). ILC2s were identified as Lineage^-^ (CD3, CD19, NK-1.1, Gr-1, CD11b, CD11c), CD45^+^, CD90.2^+^, KLRG1^+^ cells (26, 27). Flow cytometry was performed using the BD FACSLyric™ Clinical system (BD Biosciences, United States), and data were analyzed using FlowJo software (version 10.8.1). Detailed antibody information is shown in **Supplementary Table S2**.

### 2.7 Western blot

#### 2.7.1 Protein extraction and quantification

Small intestinal tissue was collected and rinsed three times with pre-chilled PBS. Tissues were minced and lysed in RIPA buffer (containing 1% PMSF and protease inhibitor cocktail) at a 1:10 (mg•μL^-1^) ratio. After homogenization on ice and shaking at 4 °C for 30 minutes, samples were centrifuged at 12,000 rpm for 15 minutes at 4 °C. Supernatants were collected and stored at -80 °C. Protein concentrations were determined using a BCA protein assay kit (Solarbio, China) (28).

#### 2.7.2 Electrophoresis and transfer

Equal amounts of protein (20 μg) were mixed with 5× loading buffer and denatured at 100 °C for 5 minutes. Proteins were separated on 10% or 12% SDS-PAGE gels (80 V for stacking, 120 V for separation) and transferred to PVDF membranes using wet transfer at 250 mA for 90–120 minutes.

#### 2.7.3 Immunoblotting and detection

Membranes were blocked with 5% non-fat milk in TBST for 1 hour at room temperature. Primary antibodies including anti-PO2F3 (1:1000), anti-DCLK1 (1:3000), anti-IL-25 (1:10000), anti-GATA3 (1:2000), anti-IL-13 (1:10000), and GAPDH (1:10000) were incubated overnight at 4 °C. After washing, HRP-conjugated secondary antibodies (1:5000) were applied for 1 hour at room temperature. Bands were visualized using ECL chemiluminescent reagent (Biosharp, China) and imaged with Azure 600 system. Band intensities were quantified using ImageJ software with GAPDH as the loading control. Detailed antibody information is shown in **Supplementary Table S3**.

### 2.8 Statistical analysis

Data are expressed as mean ± SD. The analysis was conducted with the help of GraphPad Prism software. For comparing multiple groups, we utilized one-way ANOVA, while Tukey’s test was applied for pairwise group comparisons. A p-value of less than 0.05 was deemed to indicate statistical significance.

## 3 Results

### 3.1 *Brucella* infection increases the number of intestinal tuft cells

**Figure 1:**
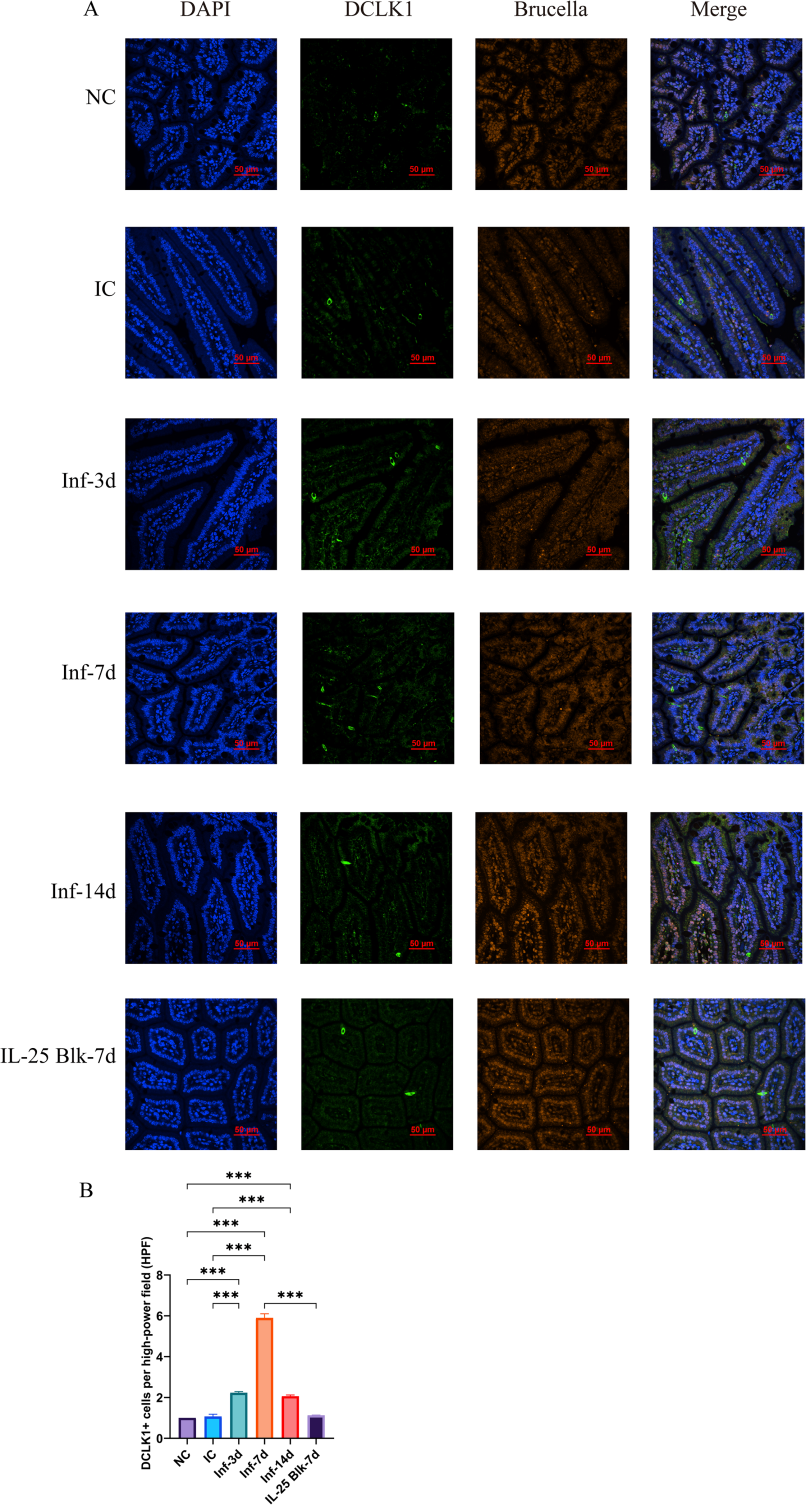
Immunofluorescence analysis of DCLK1 expression in intestinal tissues following *Brucella* infection and IL-25 blockade. **(A)** Representative immunofluorescence images showing DCLK1 (green), *Brucella* (orange) staining in intestinal tissues in different experimental groups (NC, IC, Inf-3d, Inf-7d, Inf-14d, and IL-25 Blk-7d). DAPI was used for nuclear staining (blue). Scale bar = 50 μm. **(B)** Quantification of DCLK1+ cells per high-power field (HPF) in the intestinal tissues. (Data represent mean ± SD, with statistical significance indicated by ***P<0.001.).

**Figure 2:**
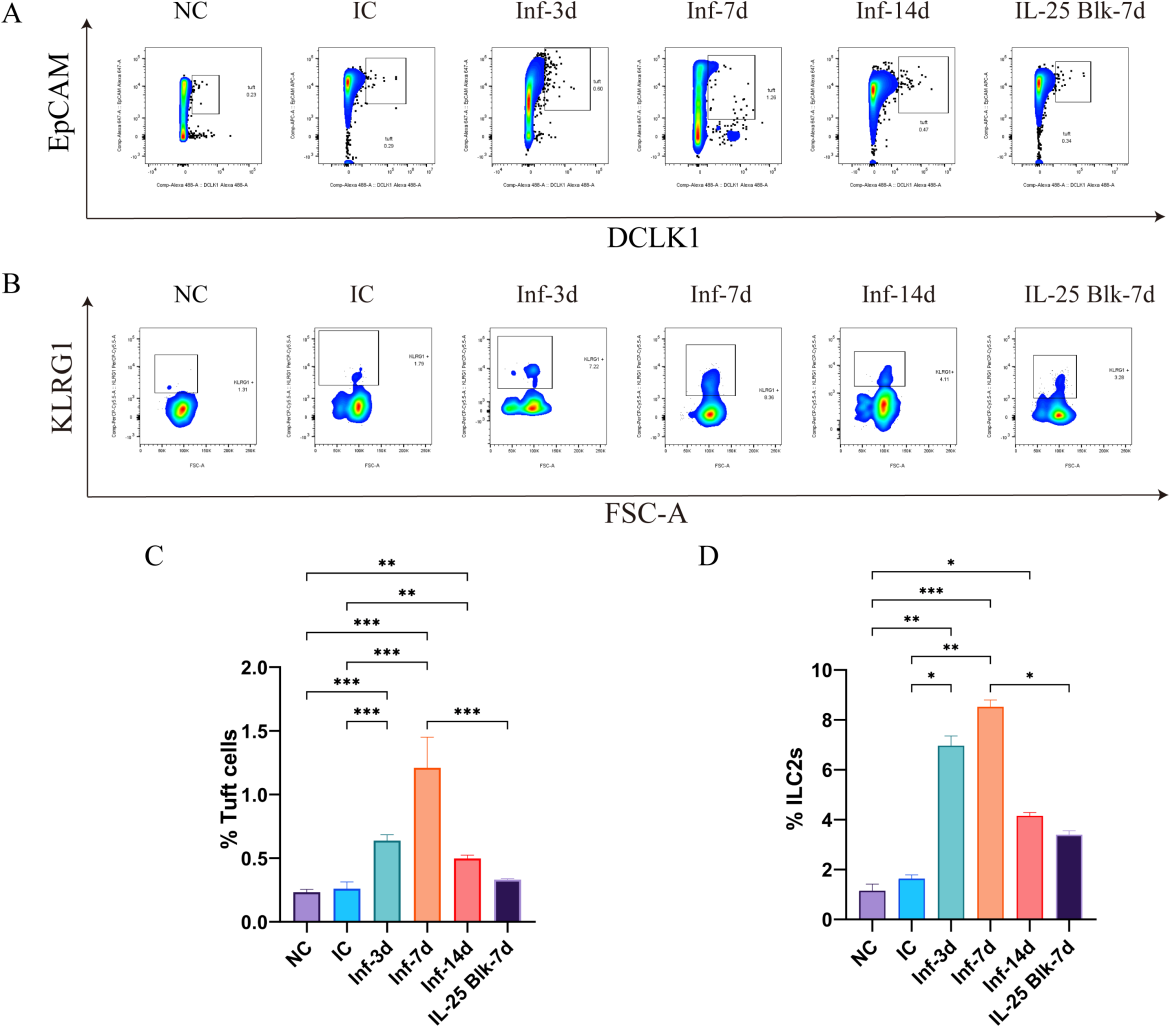
Flow cytometry analysis of tuft cells and ILC2 populations in intestinal tissues following *Brucella* infection and IL-25 blockade. **(A)** Representative flow cytometry plots of tuft cells in different experimental groups (NC, IC, Inf-3d, Inf-7d, Inf-14d, and IL-25 Blk-7d). **(B)** Representative flow cytometry plots of ILC2s in different experimental groups (NC, IC, Inf-3d, Inf-7d, Inf-14d, and IL-25 Blk-7d). **(C)** Quantification of tuft cell percentages in different experimental groups (NC, IC, Inf-3d, Inf-7d, Inf-14d, and IL-25 Blk-7d). **(D)** Quantification of ILC2 cell percentages in different experimental groups. (Data represent mean ± SD, with statistical significance indicated by *P<0.05, **P<0.01, ***P<0.001.).

To explore how *Brucella* infection alters intestinal tuft cells, we initiated our experiment by tracking the dynamic changes in DCLK1-positive tuft cells in small intestinal tissues at different stages post-infection via immunofluorescence analysis. The results showed that the number of DCLK1-positive cells in the intestines of *Brucella*-infected mice was significantly higher than those in the normal control group (NC) and the isotype control group (IC) (**Figure 1A**). Quantitative analysis revealed that the number of tuft cells began to increase at 3 days post-infection (Inf-3d) (P<0.001), peaked at 7 days post-infection (Inf-7d) (P<0.001), and remained notably elevated at 14 days post-infection (Inf-14d). Although the number of tuft cells decreased slightly at this time point, it was still significantly higher than that in the control group (P<0.001) (**Figure 1B**). Flow cytometry analysis further confirmed these findings. Tuft cells were identified as live CD45^-^EpCAM^+^ DCLK1^+^ cells in small intestinal epithelial cells (gating strategy shown in **Supplementary Figure S1**). Flow cytometry results showed that at 7 days post-infection, the proportion of tuft cells increased from approximately 0.2% in the control group to approximately 1.2%, representing a 6-fold increase (P<0.001). (**Figures 2A, C**).

### 3.2 *Brucella* infection increases intestinal ILC2 cells

To clarify the effect of *Brucella* infection on intestinal ILC2 cells, we isolated lamina propria cells from the small intestine and detected ILC2 cells by flow cytometry. ILC2s were identified as live Lin^-^CD45^+^CD90.2^+^KLRG1^+^ cells (gating strategy shown in **Supplementary Figure S2**). The results showed that *Brucella* infection induced expansion of intestinal ILC2 cells. At 3 days post-infection, the proportion of ILC2 cells began to rise from approximately 1% in the control group, peaked at day 7 at over 8%, representing an 8-fold increase (P<0.001). At 14 days post-infection, the proportion of ILC2 cells remained at a relatively high level (approximately 4.0%) (P<0.05). (**Figures 2B, D**) The expansion dynamics of ILC2 cells were highly consistent with the increasing trend of tuft cells, both peaking at 7 days post-infection.

### 3.3 IL-25 blockade inhibits the expansion of tuft cells and ILC2 cells

In order to assess IL-25’s function in the *Brucella* infection-induced tuft-ILC2 circuit, we administered anti-IL-25 neutralizing antibody to mice 24 hours before infection. The immunofluorescence findings revealed a notably decreased count of DCLK1-positive cells in the intestines of the IL-25 blockade group (IL-25 Blk-7d), which was substantially fewer than the infection-only group (Inf-7d) (P<0.001). Moreover, these cells were just slightly more abundant than those in the normal control group (P>0.05). (**Figures 1A, B**) Flow cytometry analysis revealed that IL-25 blockade significantly inhibited infection-induced expansion of tuft cells and ILC2 cells. In the IL-25 blockade group, the proportion of tuft cells decreased to approximately 0.4%, only one-third of that in the infection-only group (P<0.001). (**Figures 2A, C**) Similarly, the proportion of ILC2 cells also decreased from 8% in the infection group to approximately 3% (P<0.05). (**Figures 2B, D**) These results evidence that *Brucella* infection-induced expansion of tuft cells and ILC2 cells is dependent on IL-25-mediated mechanisms.

### 3.4 IL-25 mediates upregulation of tuft-ILC2 circuit-related protein expression

**Figure 3:**
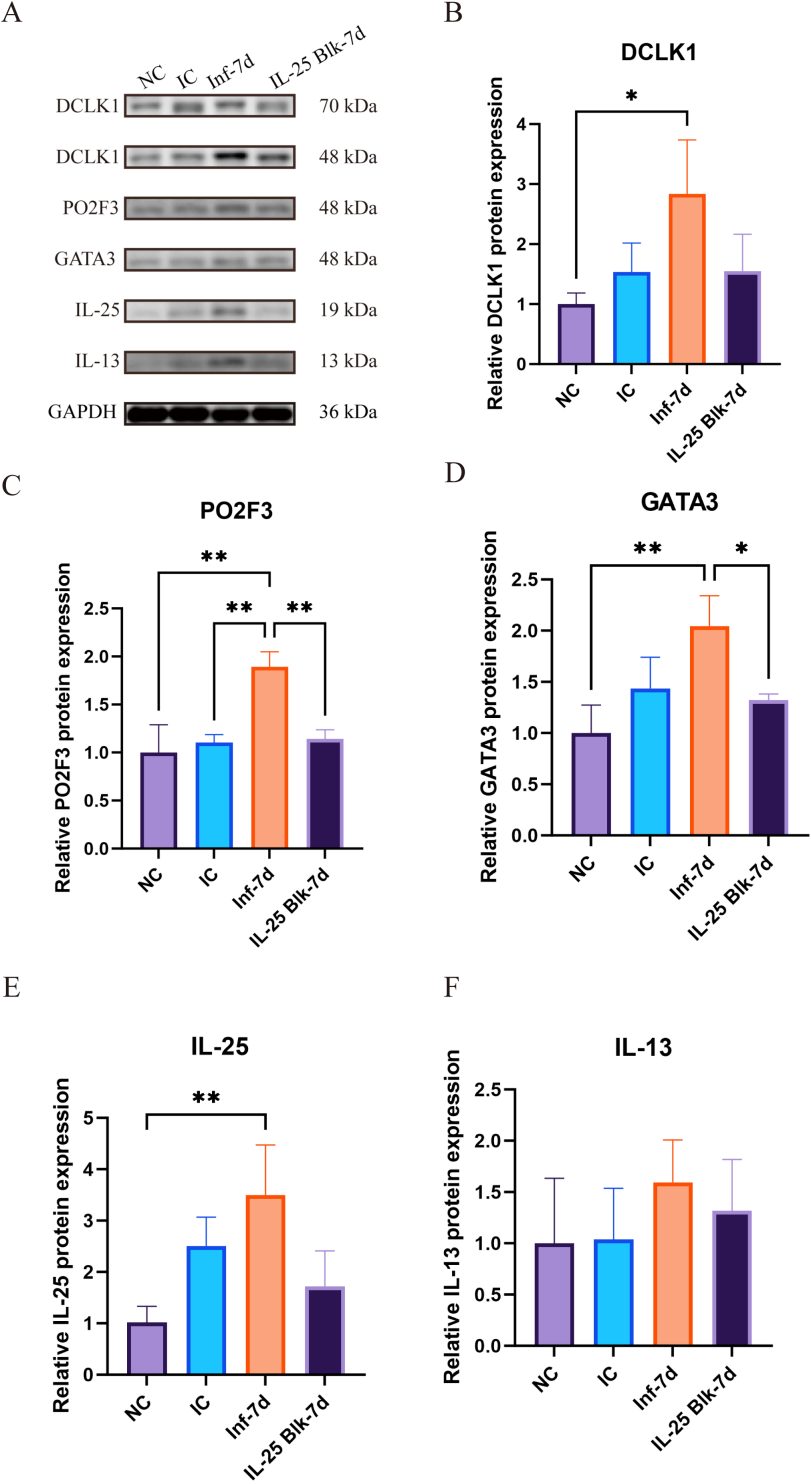
Dynamic changes in tuft cell-ILC2 circuit proteins in intestinal tissues following *Brucella* infection and IL-25 blockade. **(A)** Representative Western blot images showing the expression of tuft cell-associated proteins (PO2F3, DCLK1, IL-25) and ILC2-associated proteins (GATA3, IL-13) in small intestinal tissues from different experimental groups (NC, IC, Inf-7d, and IL-25 Blk-7d). GAPDH was used as the loading control. **(B-F)**. Densitometric quantification of relative protein expression levels of DCLK1 **(B)**, PO2F3 **(C)**, GATA3 **(D)**, IL-25 **(E)**, and IL-13 **(F)**. Protein expression was normalized to GAPDH and expressed relative to the NC group. (Data represent mean ± SD, with statistical significance indicated by *P<0.05, **P<0.01.).

To validate the above findings at the molecular level, we examined the expression changes of crucial proteins within the tuft-ILC2 circuit by Western blot (**Figure 3A**). The results clearly demonstrate that following *Brucella* infection, there was a notable increase in the levels of tuft cell-specific proteins, including PO2F3, DCLK1, and IL-25. Among these, DCLK1 protein expression increased approximately 2.8-fold compared to controls by day 7 post-infection (P<0.05) (**Figure 3B**), transcription factor PO2F3 expression increased by approximately 1.8 times (P<0.01) (**Figure 3C**), and IL-25 protein expression increased by approximately 3.5-fold (P<0.01) (**Figure 3E**). The detection results of ILC2-related proteins showed that the transcription factor GATA3 increased significantly after infection, reaching approximately twice the baseline level by day seven (P<0.01) (**Figure 3D**). Although the effector cytokine IL-13 showed an upward trend after infection, this change did not reach statistical significance (P>0.05) (**Figure 3F**). The large fluctuations in IL-13 expression may be related to differences in individual immune responses. Notably, treatment with IL-25 inhibitor suppressed the increase of these proteins. In the IL-25 blockade group, the expression levels of PO2F3, DCLK1, GATA3, and IL-13 decreased to levels close to the normal control group, forming a clear contrast with the simple infection group (**Figures 3B-F**). These changes at the molecular level also confirm the central role of IL-25 in regulating the tuft cell-ILC2 circuit.

### 3.5 Strong positive correlation between tuft cells and ILC2s is mediated by IL-25

**Figure 4:**
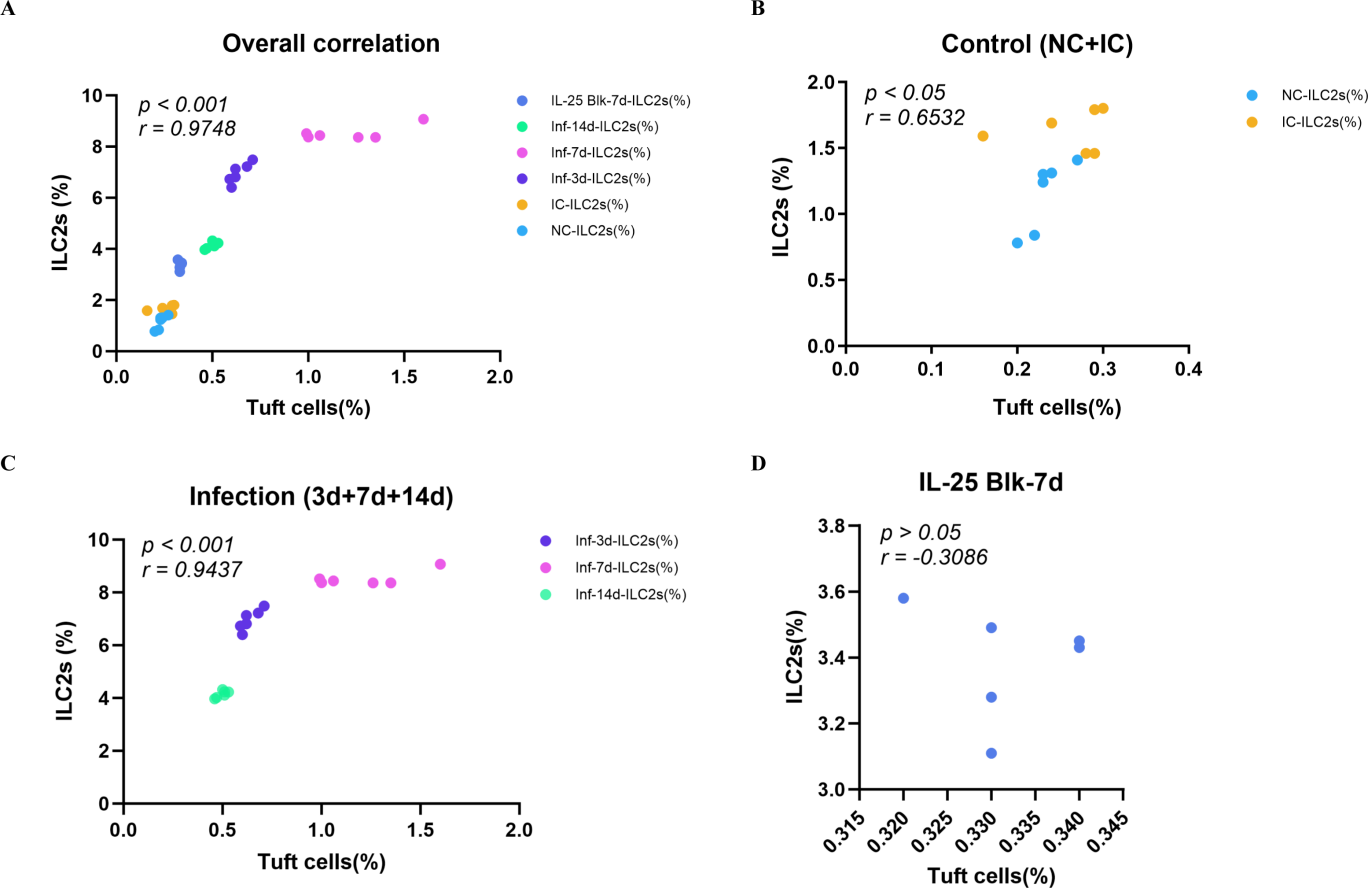
Correlation analysis between tuft cell and ILC2 percentages in intestinal tissue. Correlation analysis between tuft cell and ILC2 percentages was performed using flow cytometry data. **(A)** Overall correlation across all groups (r = 0.9748, p < 0.001, total n = 36). **(B)** Correlation in control groups (NC + IC) (r = 0.6532, p < 0.05, n = 12). **(C)** Correlation in the combined infection groups (3d, 7d, and 14d post-infection) (r = 0.9437, p < 0.001, n = 18). **(D)** Correlation in the IL-25 blocking group at 7 days post-infection (r = -0.3086, p > 0.05, n = 6). Each symbol represents an individual mouse.

In order to elaborate on the connection between tuft cells and ILC2 cells in *Brucella* infection, we conducted a correlation analysis of the flow cytometry data of each experimental group. Out of the total number of samples (n=36), tuft cell percentages and ILC2 cell percentages showed a very strong positive correlation with correlation coefficient = 0.9748 and P<0.001 (**Figure 4A**). Such close correlation signifies that there is communication between these two types of cells in the intestinal mucosa. The outcome of the analysis conducted under various conditions of the experiment reveals apparent differences. A moderate level of association was also found in the combined control groups (normal control and isotype control) with 12 individuals where the correlation coefficient stood at 0.6532 which was significant (**Figure 4B**). The correlation coefficient increased significantly to 0.9437 in the combined infection cases (3 days, 7 days and 14 days after infection) (n=18) (**Figure 4C**). This proves that *Brucella* infection enhances the linkage between the two types of cells. In the IL-25 blockade group, the correlation between tuft cells and ILC2s was abolished (r = -0.3086, not statistically significant; **Figure 4D**).

## 4 Discussion

The tuft cell-ILC2 pathway received intensive research in the context of helminth infection. Howitt and colleagues demonstrated that gustatory receptor-based sensing mechanisms by intestinal tuft cells regulate type 2 immunity against parasites (10). These basic studies however concentrated on multicellular eukaryotic parasites. von Moltke et al. and Gerbe et al. independently established that IL-25 secreted by tuft cells was a requirement in the activation of ILC2 and the expulsion of parasites (11, 12). Our laboratory studies indicate that this circuit is also responsive to bacterial pathogens. Kinetics that we have observed have rapid increase periods on day 3, peak on day 7, and maintained elevation up to day 14 which are very similar to time trends reported in helminth models.

In accordance with the analysis of the temporal course, we selected day 7 as the main time point in experiments on IL-25 blockade. This was the maximum expansion of tuft cells and ILC2s, which is the maximum circuit activation. It was in this initial stage that we were able to test the extent in which our findings reveal that cellular expansion does rely on the IL-25 in the most efficient manner possible. The notable inhibition of both the groups following the neutralization of IL-25, is a good indication of the regulatory need of IL-25.

We preferred to block IL-25 instead of the exogenous administration due to a number of reasons. The IL-25 production by endogenous cytokines is also implicated in the building of the tuft cell and ILC2, and thus we could use antibodies to neutralize IL-25 in order to obtain explicit evidence that IL-25 is essential for the tuft cell and ILC2 proliferation under *Brucella* infection. In a different study, however, we would have provided exogenous IL-25, which would have been more difficult to interpret, because IL-25 not only stimulates ILC2 but also Th2 cells (29) and eosinophils (30), which might have triggered other pathways. Moreover, our Western blot data revealed that the IL-25 expression had indeed increased by 3.5 times following infection, indicating that the *Brucella* infection model alone is a model that generates an environment of increased IL-25. The condition of infection itself is physiologically important as a functional acquisition condition. In this way, we were able to establish the need in IL-25 but eliminate some confounding factors in terms of super-physiological levels of cytokines.

There is one observation of the IL-25 blockade experiments that deserves attention. Notably, although the proportion of ILC2s in the blockade group at 7 days post-infection was lower than that in the infection group, it was still slightly higher than that in the isotype control group. This finding implies that ILC2 can be activated by IL-25-independent processes upon *Brucella* infection. We postulate that the possible pathways are the direct perception of molecular patterns by ILC2s through Toll-like receptors (31), regulating the signals through other epithelial-derived alarmins like IL-33 (32) or cysteinyl leukotrienes (cysLTs) (33), and the indirect stimulation which is mediated by inflammatory cytokines. These observations in brief demonstrate a multifaceted nature of regulations that govern ILC2s. Although IL-25 is a requisite in activating this pathway, other signaling pathways can act as backup to maintain some of the functional capabilities of ILC2s when one signaling pathway is inhibited.

In an attempt to explain the correlation of tuft cells and ILC2s, correlation analysis across all the experimental groups was conducted. There was considerable positive correlation across all groups. The correlation coefficient in the infected state was higher by far than the correlation coefficient in the control group showing that the *Brucella* stimulation has the ability of stimulating the activation level of this pathway. Interestingly, this correlation totally vanished after the neutralization of IL-25 which offers the statistical confirmation the effect of IL-25 as the mediator of the coordinated growth of these two cell groups rather than the autonomous reactions to bacterial stimulation. We admit that some mechanistic cell studies, e.g. *in vitro* co-culture studies, may assist us in learning more about this pathway. The application of such assays has however remained impractical due to the technical constraints. The proportion of intestinal tuft cells in total epithelial cells is 1.2 percent, and so it is extremely difficult to obtain enough viable tuft cells to perform *in vitro* experiments; particularly in infected tissues where the viability of the cells can be lost. As opposed to most other types of immune cells, no established tuft cell and ILC2 cell lines can reliably recapitulate the functions of the primary cells. Intestinal organoid culture systems that can promote tuft cell differentiation have already been described, but they demand special technical expertise, careful optimization of culture conditions and large-scale validation experiments to establish. These demands are beyond the available resource and the project schedule of the current research paper. These limitations influenced our choice of a study approach that involved the use of correlation analysis and *in vivo* analysis of antibody blockade to understand the functional interactions among tuft cells, IL-25, and ILC2s in the intact tissue microenvironment. There are a number of methodological weaknesses to this study, which are worth discussing. Even though the critical functional importance of IL-25 in the activation of circuits has been clearly established by antibody-mediated IL-25 neutralization, genetic methods, including the production of IL-25-deficient mice or tuft cell-specific knockout models would allow more detailed mechanistic studies. The essence of this experiment was to define the activation of tuft cells and ILC2s and their reliance on IL-25, but we did not go deeper into exploring IL-13-mediated downstream effector mechanisms (goblet cell differentiation and mucus secretion). All of these scientific questions can be subject to additional research in further studies. It must also be noted that we only analyzed the initial phases of infection (until 14 days post-infection) in our time-course analysis and that the nature of this circuit responses in chronic infection is yet to be uncovered further.

In perspective, there are a number of directions that could be further examined. More heterogeneity in tuft cell and ILC2 responses could be revealed in single-cell RNA sequencing of intestinal epithelial and immune populations, and could help distinguish functionally different subsets. Spatial transcriptomics would be useful so as to map of where activation of the circuit takes place in the anatomy, i.e. is it near Peyers patches or at ileocecal junction?

The key importance of IL-25 in the orchestration of mucosal immune response indicates that regulation of the pathway could improve oral *Brucella* immunization of the intestine. Strategies to augment IL-25 signaling (including recombinant IL-25, probiotic strains to induce tuft cell activation, and small molecules that can improve IL-25 receptor signaling) could be useful in fortifying mucosal barriers of high-risk populations (veterinarians, slaughterhouse workers, people in endemic regions). On the other hand, to target therapeutic therapies, it is possible to learn how *Brucella* can suppress or avoid tuft-ILC2 responses. The learned information can also serve to guide the response to other gastrointestinal infections whose mucosal integrity of the barrier is critical.

## Data Availability

The original contributions presented in the study are included in the article/**Supplementary Material**. Further inquiries can be directed to the corresponding authors.
