# Supplementary material for: Tuft-cell-derived IL-25 regulates intestinal ILC2 in response to Brucella infection
Source: Front Immunol. 2026 Feb 6;17:1732274. doi: 10.3389/fimmu.2026.1732274 (PMC12920439; doi:10.3389/fimmu.2026.1732274)
Supplement: Supplementary file 1 [file DataSheet1.docx]

**Supplementary figure**


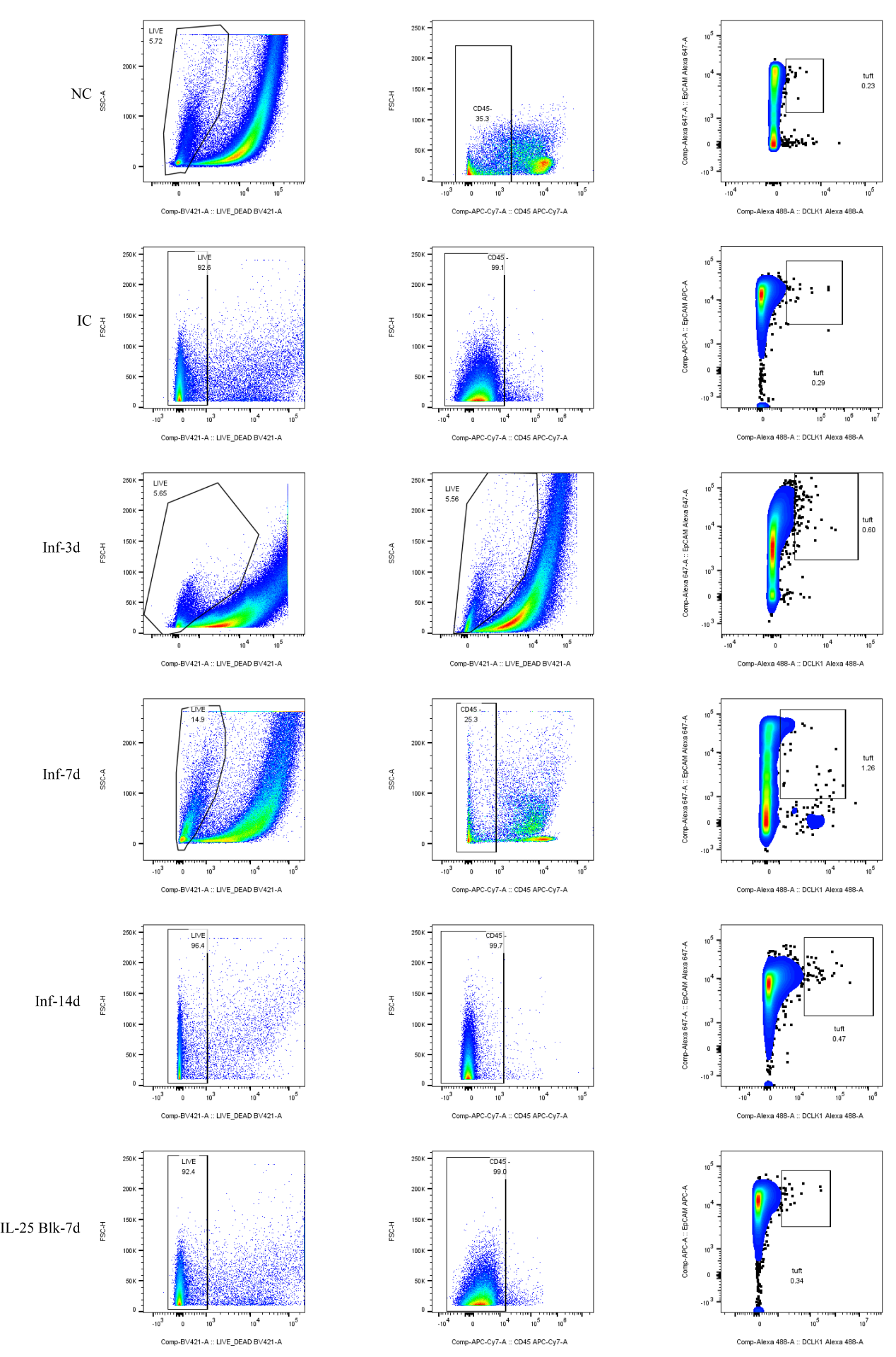


Figure S1. Representative flow cytometry gating strategy for identification of tuft cells in small intestinal epithelial cells. Cells were gated on viability (live/dead dye-), followed by CD45-, EpCAM+, and DCLK1+populations. Tuft cells were identified as viable (live/dead−), CD45−EpCAM+DCLK1+ cells. Numbers in the plots indicate the percentage of tuft cells within the gated population.


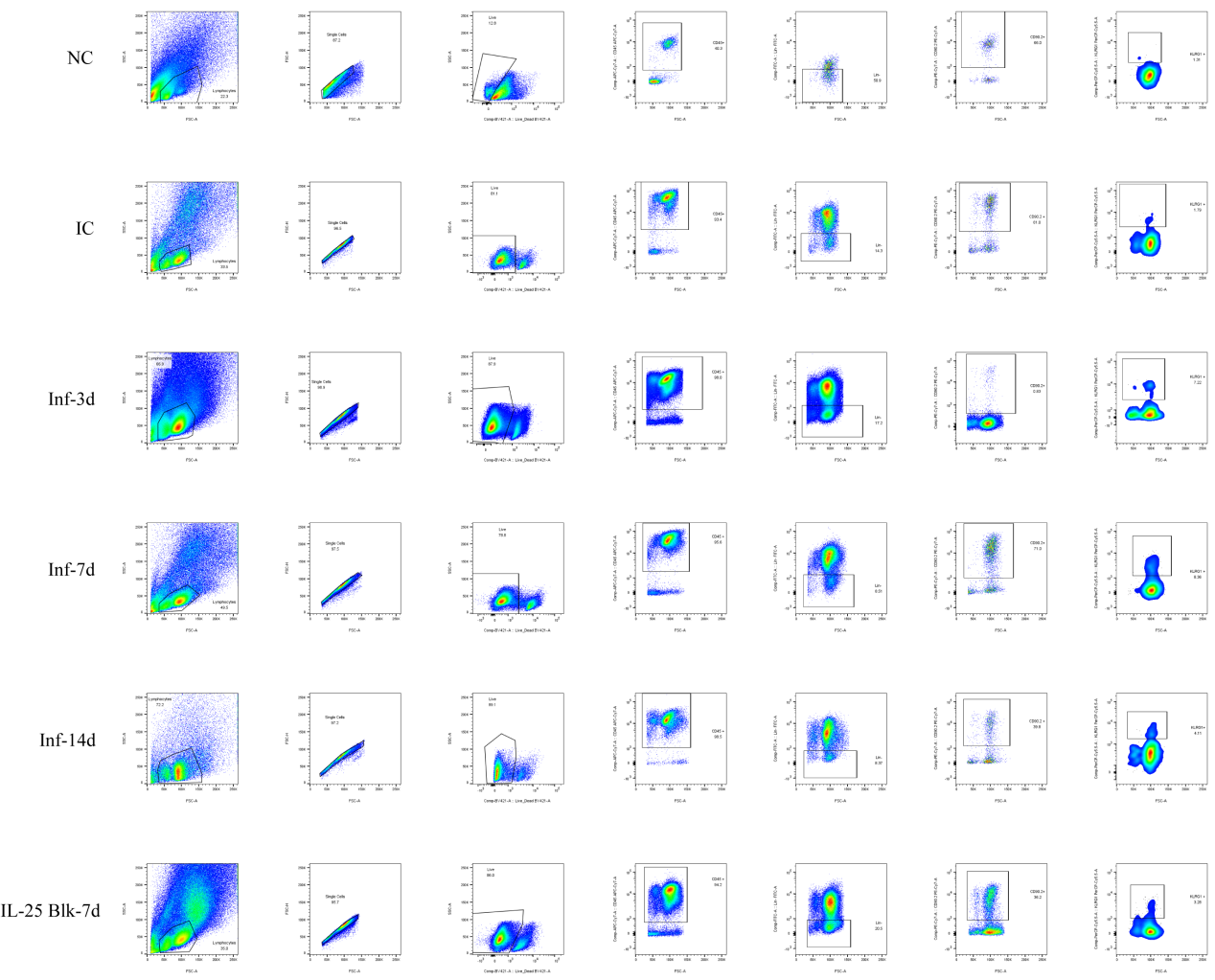


Figure S2. Representative flow cytometry plots showing ILC2 cells in small intestinal lamina propria. Cells were gated on lymphocytes (FSC-A/SSC-A), singlets (FSC-A/FSC-H), live cells, CD45+ leukocytes, lineage (CD3, CD19, NK1.1, Gr-1, CD11b, CD11c), CD90.2+, and KLRG1+ populations. ILC2s were identified as viable cells negative for lineage markers (CD3, CD19, NK-1.1, Gr-1, CD11b, CD11c) and positive for CD45, CD90.2, and KLRG1. Numbers indicate the percentage of ILC2s within the gated population.


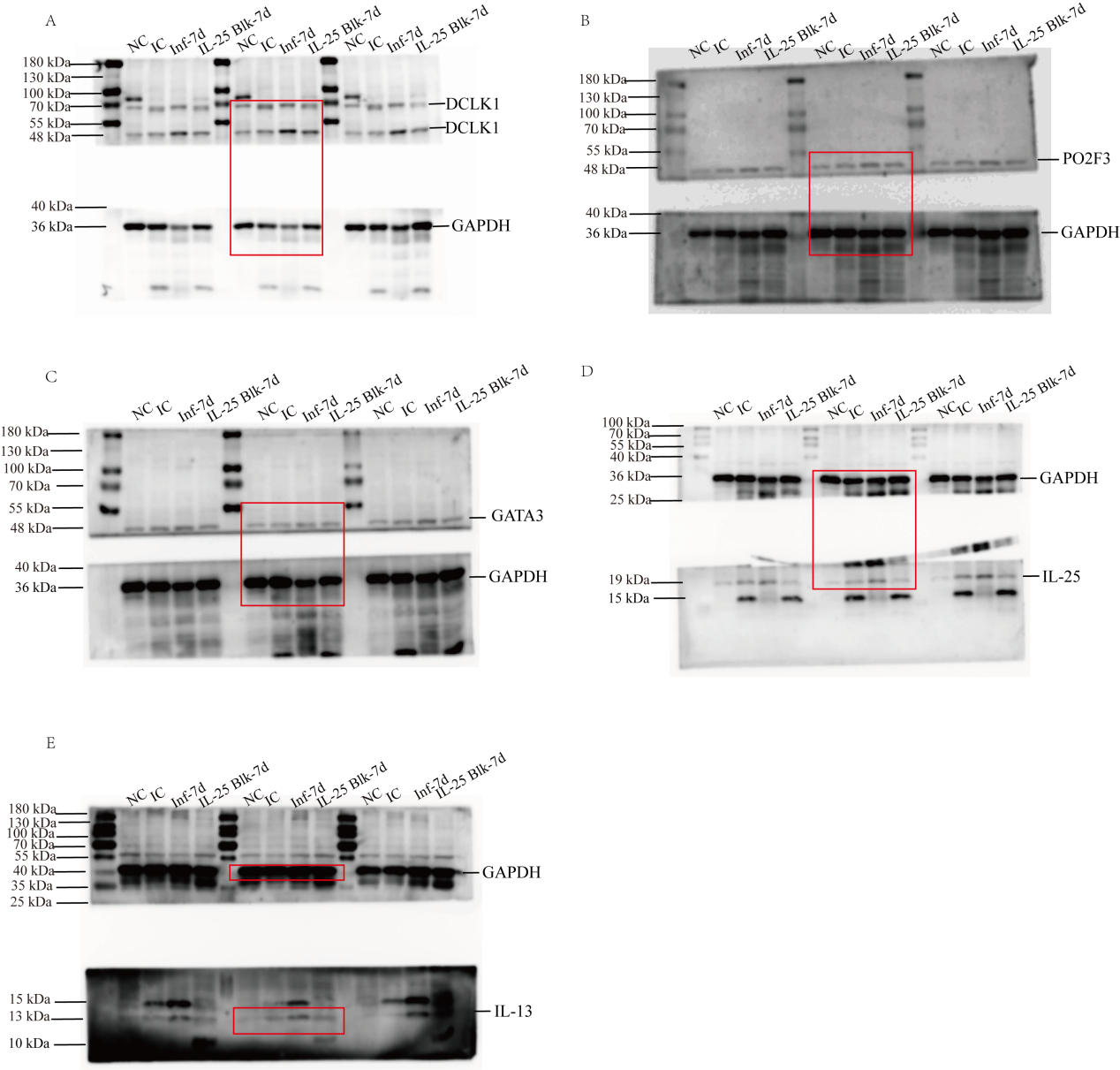


Figure S3. Original Western blot images showing protein expression in intestinal tissues following Brucella infection and IL-25 blockade.

1. DCLK1 expression with GAPDH loading control. B. PO2F3 expression with GAPDH loading control. C. GATA3 expression with GAPDH loading control. D. IL-25 expression with GAPDH loading control. E. IL-13 expression with GAPDH loading control. Lane labels: NC (normal control), IC (isotype control), Inf-3d (3 days post-infection), Inf-7d (7 days post-infection), Inf-14d (14 days post-infection), IL-25 Blk-7d (IL-25 blockade at 7 days post-infection).


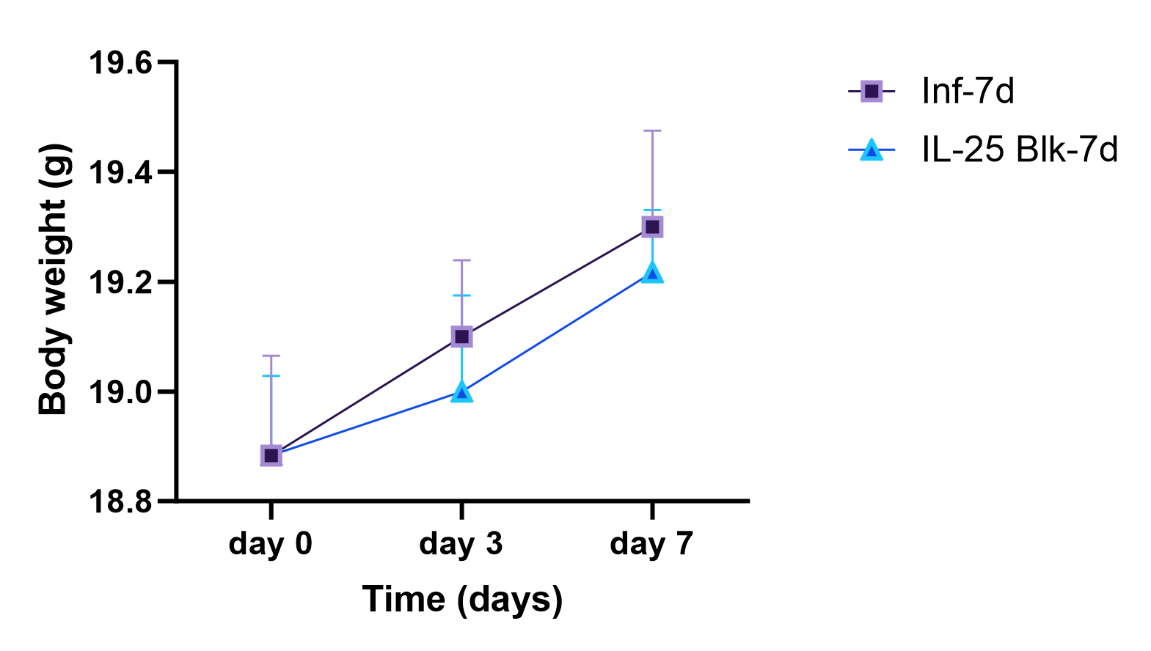


Figure S4. Body weight changes in female BALB/c mice following infection with or without anti-IL-25 antibody treatment.

Female BALB/c mice received intraperitoneal injection of anti-IL-25 neutralizing antibody (IL-25 Blk-7d, blue triangles) or were left untreated as infected controls (Inf-7d, purple squares). Body weight was measured at days 0, 3, and 7 post-infection. Data are presented as mean ± SEM.


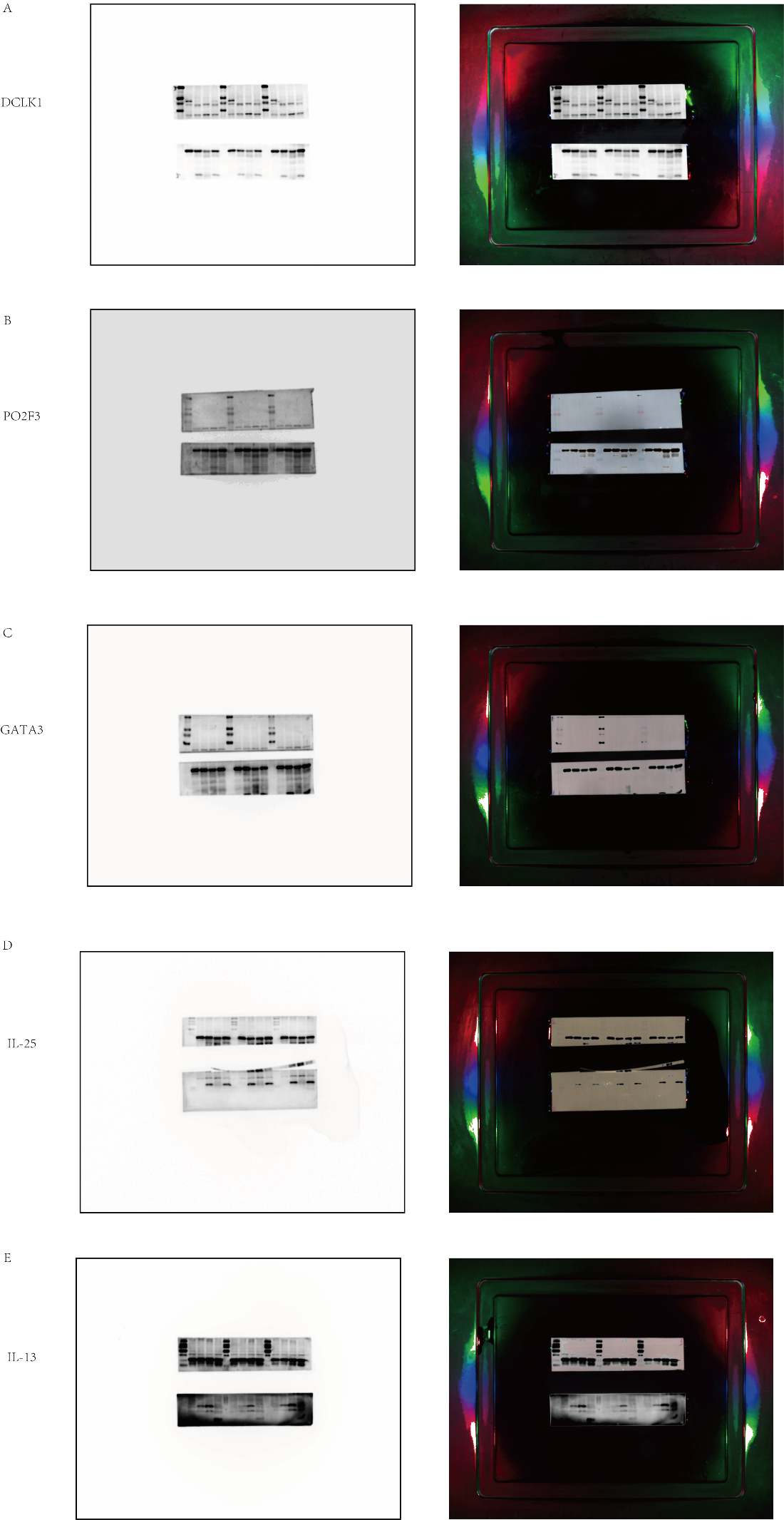


**Figure S5. Original Western blot membranes for target protein detection.**

**(A–E) Representative original Western blot results for the expression of DCLK1 (A), PO2F3 (B), GATA3 (C), IL-25 (D), and IL-13 (E). Left panels: Grayscale images of chemiluminescent signals (target protein bands) captured via imaging system. Right panels: Photographs of the physical membranes after signal development.**

**Supplementary table**

Table S1. Reagents and antibodies used for immunofluorescence.

| Antibodies | Company | Country | Catalog Number |
| --- | --- | --- | --- |
| *Brucella* Rabbit pAb | Bioss | China | bs-2229R |
| DCLK1 Polyclonal antibody | Proteintech | China | 21699-1-AP |
| Four-color multifluorescent  immunohistochemical staining kit | Absin | China | abs50028 |

Table S2. Flow cytometry staining antibodies.

| Antibodies | Company | Country | Catalog Number |
| --- | --- | --- | --- |
| DCLK1 Polyclonal antibody | Proteintech | China | 21699-1-AP |
| CoraLite488-conjugated Goat Anti-Rabbit IgG(H+L) | Proteintech | China | SA00013-2 |
| Alexa Fluor® 647 anti-mouse CD326 (Ep-CAM) Antibody | BioLegend | USA | 118212 |
| BD Pharmingen™ APC-Cy™7 Rat Anti-Mouse CD45 | BD | USA | 557659 |
| LIVE/DEAD™ Fixable Dead Cell Stain Kits | Invitrogen | USA | L34963 |
| BD Pharmingen™ PE-Cy™7 Rat Anti-Mouse CD90.2 | BD | USA | 561642 |
| PerCP/Cyanine5.5 anti-mouse/human KLRG1 (MAFA) Antibody | BioLegend | USA | 138418 |
| FITC anti-mouse CD3 Antibody | BioLegend | USA | 100204 |
| FITC anti-mouse CD19 Antibody | BioLegend | USA | 115506 |
| FITC anti-mouse NK-1.1 Antibody | BioLegend | USA | 108706 |
| FITC anti-mouse Ly-6G/Ly-6C (Gr-1) Antibody | BioLegend | USA | 108406 |
| FITC anti-mouse/human CD11b Antibody | BioLegend | USA | 101206 |
| FITC anti-mouse CD11c Antibody | BioLegend | USA | 117306 |

Table S3. Antibodies used for Western blot (WB) Experiments.

| Antibodies | Company | Country | Catalog Number |
| --- | --- | --- | --- |
| PO2F3 Rabbit pAb | immunoway | China | YT6917 |
| DCLK1 Polyclonal antibody | Proteintech | China | 21699-1-AP |
| IL-25/IL-17E Recombinant monoclonal antibody | proteintech | China | 86396-1-RR |
| GATA3 Rabbit pAb | immunoway | China | YT8149 |
| IL-13 Recombinant monoclonal antibody | proteintech | China | 83935-1-RR |
| GAPDH Polyclonal antibody | proteintech | China | 10494-1-AP |
